# A New Model to Study Fatigue in Dental Implants Based on Probabilistic Finite Elements and Cumulative Damage Model

**DOI:** 10.1155/2017/3726361

**Published:** 2017-07-05

**Authors:** María Prados-Privado, José Antonio Bea, Rosa Rojo, Sérgio A. Gehrke, José Luis Calvo-Guirado, Juan Carlos Prados-Frutos

**Affiliations:** ^1^Department of Medicine and Surgery, Faculty of Health Sciences, Rey Juan Carlos University, Madrid, Spain; ^2^University of Zaragoza, Zaragoza, Spain; ^3^Biotecnos Research Center, Montevideo, Uruguay; ^4^Faculty of Medicine & Dentistry, San Antonio Catholic University of Murcia (UCAM), Murcia, Spain

## Abstract

The aim of this study was to predict the fatigue life of two different connections of a dental implant as in load transfer to bone. Two three-dimensional models were created and assembled. All models were subjected to a natural masticatory force of 118 N in the angle of 75° to the occlusal plane. All degrees of freedom in the inferior border of the cortical bone were restrained, and the mesial and distal borders of the end of the bone section were constrained. Fatigue material data and loads were assumed as random variables. Maximum principal stresses on bone were evaluated. Then, the probability of failure was obtained by the probabilistic approach. The maximum principal stress distribution predicted in the cortical and trabecular bone is 32 MPa for external connection and 39 MPa for internal connection. A mean life of 103 and 210 million cycles were obtained for external and internal connection, respectively. Probability cumulative function was also evaluated for both connection types. This stochastic model employs a cumulative damage model and probabilistic finite element method. This methodology allows the possibility of measured uncertainties and has a good precision on the results.

## 1. Introduction

Dental implants have been widely employed to replace missing teeth and have become routine elements of dental practice [1, 2] with a success rate higher than 90% [3]. Despite this high success rate, dental implant failure can occur. Brunski detailed in [4] that how biting forces are transferred to the surrounding bone play an important role on the success or failure of a dental implant.

Load transfer from implants to bone is influenced by the type of loading, the implant geometry (length and diameter), the shape and characteristics of the implant surface, and the quantity and quality of the surrounding bone [5, 6]. A huge number of experimental and numerical studies have been implemented with the aim of understanding the mechanism of load transfer from the implants to the bone [7].

The connection of the dental implant also has influence in the load transfer and the stress distribution. Many studies have reported that the most crucial factor in dental implant fatigue is the geometry of the dental implant-abutment connection, a screw preload, dental implant fixation, and crown loading [8]. Some studies have reported mechanical complications with external hexagon connections because of the limited resistance to oblique load [9, 10]. Abutment connection design also affects the stress concentration in the surrounding bone [11].

Manufacturers have developed different types of implant-abutment connections with the aim of obtaining implant stability. For a good implantation, implants must resist the stress and transmit forces to the bone [12]. Mechanical complications have been reported to increase when external hexagon connections are used, due to their instability and reduced resistance to oblique loads.

Finite element analysis (FEA) has been extensively used in dentistry for analyzing different aspects in this field. Asmussen et al. and Maceri et al. employed this tool in different restorative techniques [13, 14]; Baggi and coworkers and Himmlová et al. used it for the influence of implant and prosthesis design [15, 16], among other authors that employed some three-dimensional finite element analyses in some of their studies.

Most of the finite element analyses, including the previous ones, were deterministic. However, in dental implant field, it is important to evaluate the impact of some factors such as geometry, loading conditions, or material properties [17] because the combination of the uncertainty of a parameter can modify the component behavior.

Dental implants and their components must support mastication forces which act on a cyclic manner, and therefore, fatigue is introduced in dental implant-supported rehabilitation. A good measure of variability and uncertainty is decisive for having more accuracy on the results because fatigue is very sensitive to many different parameters, as material properties and load history [18].

The main purpose of this study was to predict the fatigue life of different commercial dental implants and their stress transfer properties. The stochastic methodology employed here to obtain the probability of failure and the principal statistic of the fatigue life is based on a cumulative damage model and a probabilistic finite element method. The applied occlusal forces and fatigue implant material properties were the random variables. Two commercial implants with different connections have been analyzed with this methodology. The fatigue behavior, the probability of failure, and the mechanical behavior at the bone-implant interface have been evaluated.

## 2. Materials and Methods

Two dental implants were employed in this study. These implants are manufactured by Proclinic® (Avenir, Italy), and their characteristics are described in Table 1.

### 2.1. Finite Element Model

**Figure 1:**
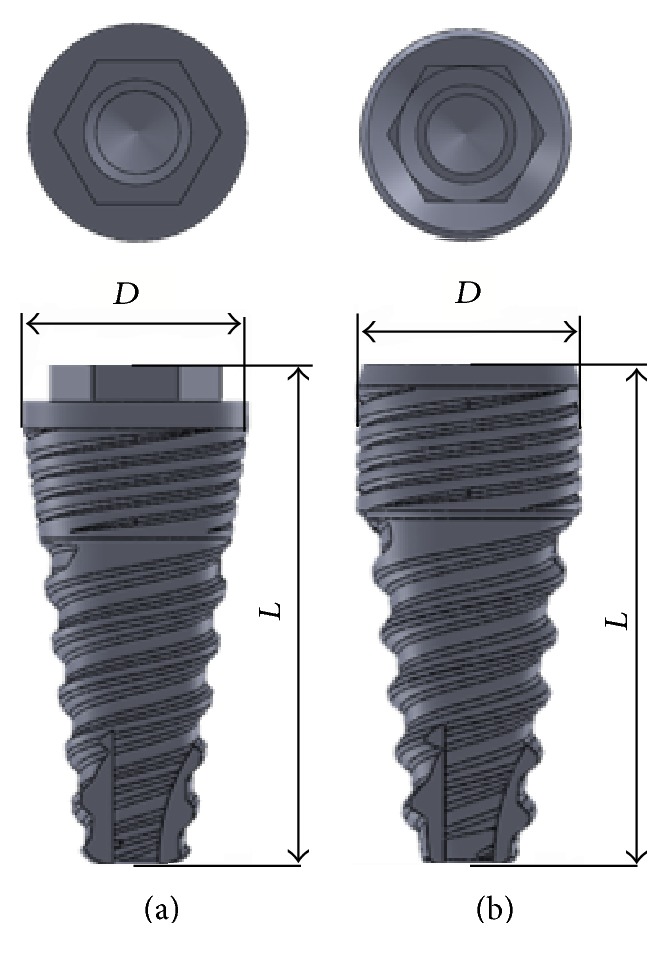
Geometry of the dental implants analyzed in this study (*L*: implant total length; *D*: diameter). (a) External connection; (b) internal connection.

**Figure 2:**
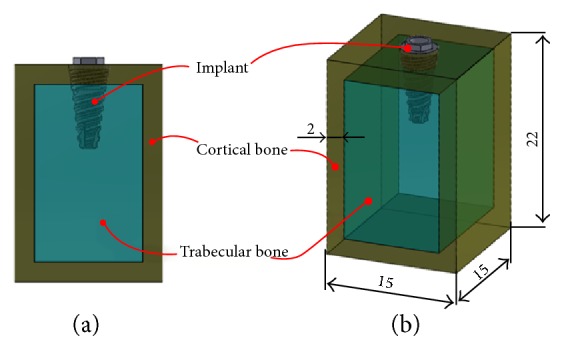
(a) Regions modelled in the finite element model of dental implants and bone. (b) Bone dimensions.

Figure 1 represents the geometry of the two dental implants employed in this study. Once the implants' geometry is defined, the surrounding bone must be represented (Figure 2(a)).

Two 3D models were created employing the CAD software Solidworks 2016 (Dassault Systemes, SolidWorks Corp., Concord, MA, USA). Dental implants were provided by the manufacturer, and bone geometries were created employing SolidWorks.

A D2 bone type [19] was simulated, and its characteristics were obtained from Vootla and coworkers [20] and are detailed in Table 2. Dimensions used in the bone geometry ensure enough distance between the implants and the ends of the model, thus avoiding any undesired boundary effects (Figure 2(b)).

Once all models were assembled, they were imported into ANSYS Workbench 16 (Canonsburg, PA, USA) and analyzed. An adequate finite element mesh was crucial in this problem due to stress singularities expected at the sharp corners. The convergence criterion was a change of less than 5% in von Mises stress in the model [21]. The number of elements and nodes employed in this study is summarized in Table 3.

### 2.2. Material Properties

Implants were made from Ti6Al4V. The elastic properties of the titanium alloy and bone used in the models were taken from the literature. Implants and bone were modelled with linear, elastic, isotropic, and homogeneous properties [6]. The elastic modulus and Poisson's ratio of the titanium alloy were 100 GPa and 0.3, respectively [22]. Bone properties were taken from [23], and they are summarized in Table 4.

The ultimate stress of cortical bone has been reported to be higher in compression (170 MPa) than in tension (100 MPa) [24]. The strength of the trabecular bone has been reported to be the same in tension and compression and is approximately 2–5 MPa [24].

### 2.3. Boundary Conditions and Loading Configuration

**Figure 3:**
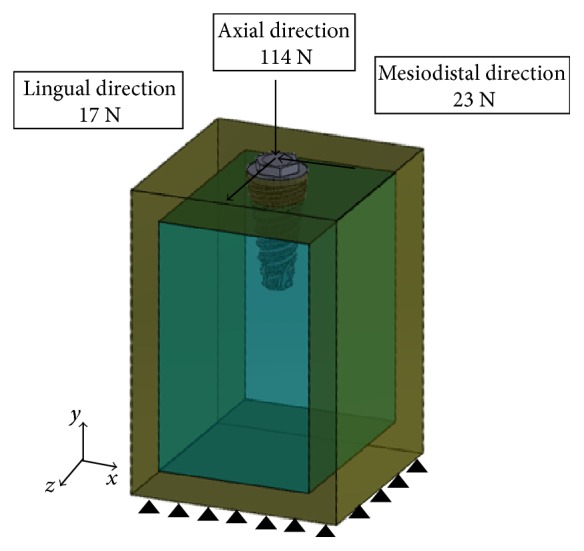
Applied loads and boundary conditions.

All models employed in the present study were constraint as detailed: all degrees of freedom in the inferior border of the cortical bone were restrained, and the mesial and distal borders of the end of the bone section were constrained so that the displacement of nodes in the direction perpendicular to the surface was equal to zero (Figure 3). For simulating osseointegrated condition, the implants were rigidly bonded in the bone.

An average physiological bite force was simulated (114.6 N in the axial direction, 17.1 N in the lingual direction, and 23.4 N toward the mesial at an angle of 75° to the occlusal plan) (Figure 3) [25].

### 2.4. Probabilistic Fatigue Model

In addition to the previous deterministic FE analysis, a probabilistic fatigue model is also implemented. Although fatigue phenomenon has a probabilistic nature which can compromise the usefulness and validity of the system, most of the studies available in the literature have been done from a deterministic point of view.

Random variables considered in this study have been Young's modulus and mastication loads due to its influence on the life of the structural components [26].

**Figure 4:**
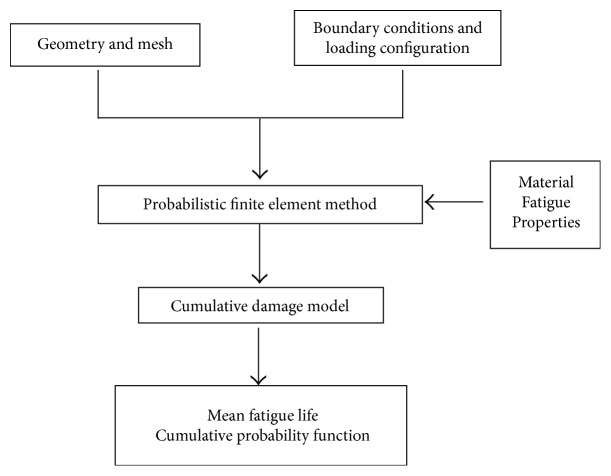
Scheme of the probabilistic model.

A schematic summary of the probabilistic methodology is shown in Figure 4.

Firstly, the cumulative damage model (B-model) must be defined [27]. This model requires to define the statistic characteristics (mean and variance) of the variables involved in the damage process (stresses/strains and material properties).

Authors have employed a cumulative damage model, called B model, based on Markov chains and developed by Bogdanoff and Kozin [27]. The hypotheses that serve as a basis for the expansion of the B-unit step model are the following:

1. Damage cycles (DC) are repetitive and of constant severity.
2. The levels of damage a component will go through until final failure are discrete (1, 2,…, j,…, b), and failure occurs at the last level of damage (b). This hypothesis merely discretizes the total life of the component in b levels.
3. The accumulation of damage that occurs in each DC depends only on the DC itself and the level of damage of the component at the start of the said DC.
4. The level of damage in each DC can only be increased from the occupied level at the beginning of the said DC to the next immediate level.

The mathematical formulation of these hypotheses is developed below. Vector **p**_0_ is defined as the initial distribution of damage levels for *x* = 0:

(1) $$\begin{array}{c} p_{0}=\left\{\pi_{1},\pi_{2},\ldots ,\pi_{b-1},0\right\}, \end{array}$$

verifying that

(2) $$\begin{array}{c} \overset{b-1}{\underset{j=1}{\sum }}\pi_{j}=1. \end{array}$$

According to hypothesis 1, damage cycles have been defined as constant severity, so the probability transition matrix (**P**) will expresses the probability that each DC has to be in the same level or the probability of jump to the next DC [28]:

(3) $$\begin{array}{c} P=\left(\begin{array}{ccccccc} p_{1} & q_{1} & 0 & 0 & \ldots & 0 & 0\\ 0 & p_{2} & q_{2} & 0 & \ldots & 0 & 0\\ 0 & 0 & p_{3} & q_{3} & \ldots & 0 & 0\\ \vdots & \vdots & \vdots & \vdots & \ddots & \vdots & \vdots \\ 0 & 0 & 0 & 0 & \ldots & p_{b-1} & q_{b-1}\\ 0 & 0 & 0 & 0 & \ldots & 0 & 1 \end{array}\right). \end{array}$$

Due to damage cycles that have been defined as constant severity, the probability transition matrix (**P**) will be unique.

Using the results of Markov chains, vector **p**_*x*_ is

(4) $$\begin{array}{c} p_{x}=p_{x-1}P=p_{0}P^{x},\;con\;x=0,1,2,\ldots . \end{array}$$

The expected value and variance of *N_f_* are obtained as detailed:

(5) $$\begin{array}{c} E\left[N_{f}\right]=\overset{b-1}{\underset{j=1}{\sum }}\left(1+r_{j}\right),\\ \mathrm{var}\left[N_{f}\right]=\overset{b-1}{\underset{j=1}{\sum }}r_{j}\left(1+r_{j}\right), \end{array}$$

with *r*_*j*_ = *p*_*j*_/*q*_*j*_ and *p*_*j*_ = *r*_*j*_/(1 + *r*_*j*_), where *p*_*j*_ is the probability of remaining in the same DC and *q*_*j*_ is probability of jumping to the next DC.

In the present work, fatigue in random crack stage is analyzed. With this goal, expressions as those of Neuber, Ramberg-Osgood, and Coffin-Basquin-Manson must be employed with the aim of obtaining the statistical estimators.

The relation between the elastic stress and strain is expressed by Neuber's rule [29, 30] and detailed in

(6) $$\begin{array}{c} \frac{\sigma_{ep}}{E}+{\left(\frac{\sigma_{ep}}{k}\right)}^{1/n^{\prime }}-\frac{\varepsilon_{el}^{2}\cdot E\;}{\sigma_{ep}}=0,\\ \sigma_{el}=E\cdot \varepsilon_{el}, \end{array}$$

where *ε*_el_ is the elastic strain, *σ*_el_ is the elastic stress, *k* is the strength coefficient, and *n*′ is the strain-hardening exponent

As it is detailed in [28], the estimators of elastic-plastic deformations and stresses have been obtained by the use of Neuber's rule. The expected values and variances of von Mises strain and stress were obtained from the probabilistic finite element analysis.

Then, the expected values and variances of the elastic-plastic magnitudes involved in the formulation of Coffin-Basquin-Manson, from a linear static analysis using Neuber's correction, must be obtained [31–34]. Coffin and Basquin proposed a nonexplicit relationship between the fatigue life cycles in the nucleation stage of a component and the amplitudes of strain:

(7) $$\begin{array}{c} \frac{∆\varepsilon_{ep}}{2}=\frac{{\sigma_{f}}^{\prime }}{E}{\left(2N_{f}\right)}^{b}+{\varepsilon_{f}}^{\prime }{\left(2N_{f}\right)}^{c}, \end{array}$$

where ∆*ε*_ep_ is range of elastic-plastic strain suffered by the component at the crack initiation area, *σ*_*f*_′ is fatigue resistance coefficient, *ε*_*f*_′ is fatigue ductility coefficient, *b* is fatigue resistance exponent, *c* is fatigue ductility exponent, *E* is modulus of elasticity, and *N*_*f*_ is fatigue life cycles.

In the current study, the probabilistic finite element method has been used to obtain the principal statistics of the response variables of the system with respect to the random variables introduced as data.

To develop this model, the stochastic values (mean and standard deviation) of the material properties and loads should be known (Table 5).

Once FE models analyzed, the random distribution (mean and variance) of stress and strains in implants is evaluated by the probabilistic finite element method, which avoids a Monte Carlo simulation [35]. The reader is referred to Prados-Privado et al. [28] for further details.

The aim of the model is to calculate the fatigue life of the component studied. To compute this random variable is necessary to use the damage model, which is based on Markov chains [27]. The probabilistic transition matrix (PTM) can be obtained from the computed mean value and variance of the fatigue life, and from this PTM, it is possible to obtain the probability of failure of the implant [28].

## 3. Results and Discussion

This paper applies a probabilistic methodology for two titanium dental implants, considering the variability in loads and Young's modulus. This method can be employed as a systematic technique to determine the effect of uncertainties of mechanical factors in the performance. The method proposed here was validated in [36–38].

Due to the uncertainties between bite habits among different patients, loads cannot be considered as deterministic. This model considers these uncertainties from the very beginning. Limitations of this method are mainly related to the coefficient of variation of all the random variables involved. As far as we use first order Taylor expansions, the spread of every random variables cannot be wide. A second order or a different approach must be used in this last case. Most FE studies on dental implants and pieces need to place them are static analyses [39, 40].

The first step to construct the model employed in this work is the probabilistic model developed by Bogdanoff and Kozin, considering as random most of the variables involved. Then, the finite element method and the B-model are used to solve the mean values for the probabilistic problem and the variance of them.

Photoelasticity and finite elements are two tools which have been employed to a better understanding of the stress transfer and distribution from implants to surrounding bone [35]. In general, dental implants should be dared to distribute properly the loads with a nonexcessive concentration area, and if excessive stress is applied to bone, bone resorption can occur.

Several studies have analyzed the fatigue failure of dental implants [10, 41]. Cycled loads applied result in strains and micromotions that can introduce fatigue failure of the dental implant [42]. Bite habits also causes different loads on the implants. Therefore, the analysis of dental implants presents clear stochastic characteristics, requiring a probabilistic approach as the ones detailed in this study.

The stress field on implants (von Mises stress) and surrounding bone (maximum principal stress) was evaluated for the case of previous static loading. The maximum principal stress distribution predicted in the cortical and trabecular bone is 32 MPa for model number 1 and 39 MPa for model number 2. These values are considerably lower than ultimate stress values in tension (approximately 100 MPa).

Load transfer mechanisms have been studied from many years helping to increase the success rate [43, 44]. Santiago et al. detailed in [43] that there is no consensus about the connection of dental implants although some studies have associated the external hexagon with higher bone loss rates. It is important to know stress distribution on dental implants because it is possible to predict where the fracture or failure will occur.

With the aim of reproducing realistic loads in dental implant environment, a combined force must be applied in a finite element analysis to dental implants [45]. An average bite force in a natural and oblique direction was applied in the present study.

The highest von Mises stress in both dental implants appears around the neck of the implants, which is in accordance with the literature [46]. In this case, stress in implants varying from 252 MPa in model number 1 (Figures 5(a) and 5(b)) to 109 MPa in model number 2 (Figures 5(c) and 5(d)). These values are lower than yield stress in the titanium alloy (around 650 MPa) [6]. In addition to this, stresses are spread from the neck to the apical area where stress is minimum (Figure 5).

The probabilistic methodology proposed was employed to estimate the principal statistics of the fatigue life (mean and variance) and the probability of failure of these two dental implants.  Table 6 details the principal statistics obtained for each dental implant. These values have been obtained at the most critical point that appears when the load described in Figure 3 is applied.

**Figure 6:**
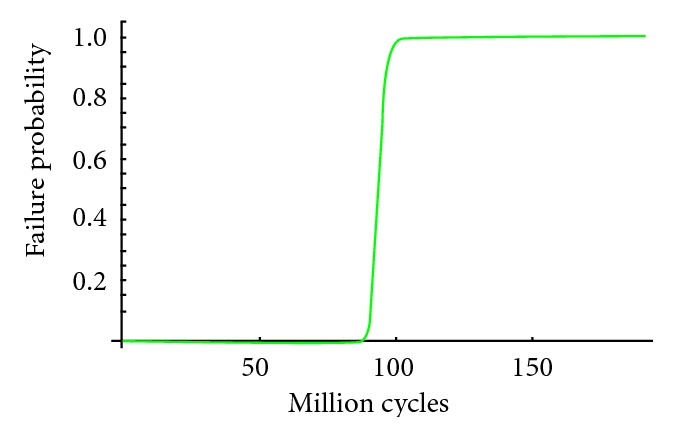
Probability of failure function for external dental implant.

**Figure 7:**
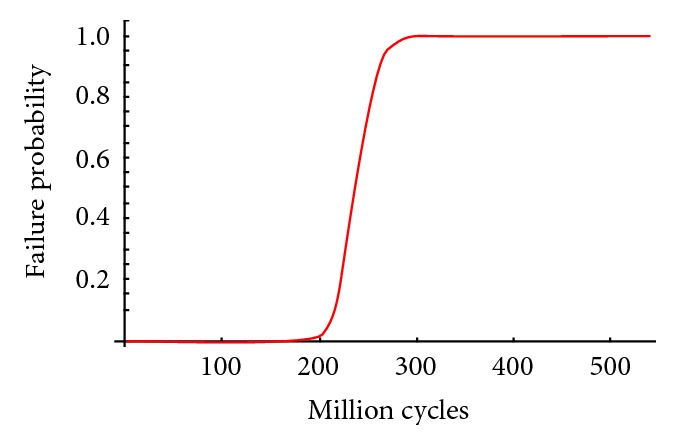
Failure probability function for internal dental implant.

We are able to determine the failure probability of these two dental implants analyzed for a specific number of load cycles. The probability of failure associated with each cycle was obtained for the maximum stress. The evolution of the probability of failure was evaluated from ten million loading cycles to 200 million loading cycles for model number 1 (Figure 6) and from ten to 550 million cycles for model number 2 (Figure 7). Gibbs et al. defined one million loading cycles as about one year of in vivo service [47].

For a fixed number of loading cycles, different failure probabilities were predicted for each commercial implant. Failure probability diagram relates the probability of failure associated with each cycled load.

Fatigue phenomenon in dental implants is very sensitive to uncertainties in variables involved in this phenomenon, but despite this, most of the studies available in the literature have been done from a deterministic point of view [16, 48]. Mathematical bone employed in this study could help to understand and to improve the task of dental implant design and their failure rates. In that sense, this probabilistic model can determine the influence of many variables that take part on dental implant fatigue phenomenon.

## 4. Conclusions

This study has applied a probabilistic methodology to two commercial implants with the aim of evaluating the effect of the connection in the mean life and the probability of failure. The current approach is based on Markov chains, cumulative damage model (B-model), and the probabilistic finite element analysis. Load transfer to a D2 bone in both dental implants has been also evaluated. Our results show that for a D2 bone, internal connection is more effective on distributing loads than external hexagon.

An implant with the internal connection has a better fatigue behavior because a bigger mean life was obtained and, therefore, a better cumulative probability function.

## Figures and Tables

**Figure 5:**
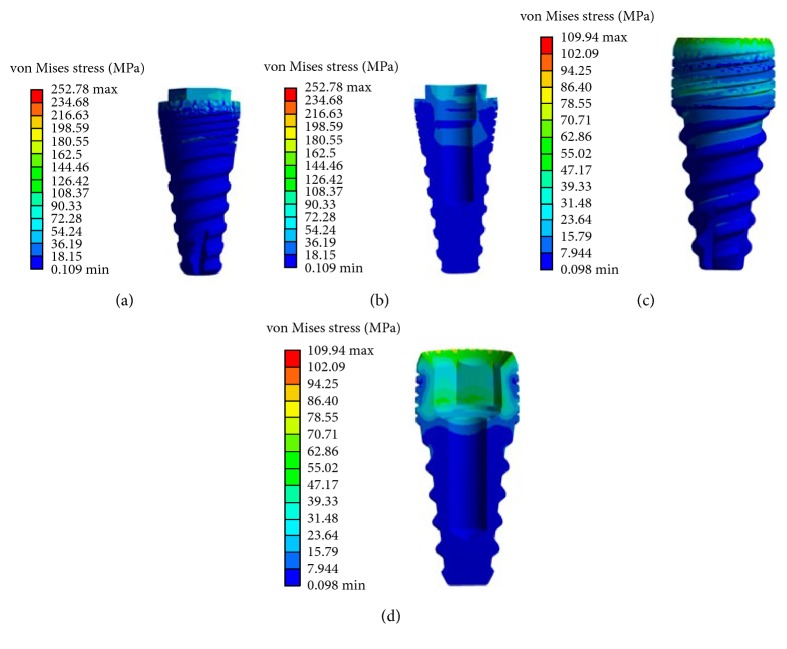
Stress distribution in dental implants. (a) Model number 1. (b) Cross section of model number 1. (c) Model number 2. (d) Cross section of model number 2.

**Table 1:** Dental implant characteristics.

Model number (catalogue name)	Implant morphology	Connection	Implant diameter × length (mm)
1 (IP851)	Conical	External hexagon	3.5 × 8
2 (IP876)	Conical	Internal hexagon	3.5 × 8

**Table 2:** Geometrical dimensions for cortical bone model.

Cortical thick (mm)	Height (mm)	Width (mm)
2	22	15

**Table 3:** Number of nodes and elements in each FE model.

	Nodes	Elements
Model number 1 (external hexagon)	733,147	513,285
Model number 2 (internal hexagon)	1,058,362	748,113

**Table 4:** Cortical and trabecular bone properties.

	Young's modulus (GPa)	Poisson's ratio
Cortical bone	13.7	0.3
Trabecular bone	4	0.3

**Table 5:** Stochastic values of the material properties and loads.

Mean mastication load ± standard deviation (N)	Titanium Young's modulus ± standard deviation (GPa)
118 ± 30	100 ± 20

**Table 6:** Mean and variance of the fatigue life for each dental implant.

	Mean life (million cycles)	Variance (million cycles^2^)
Model number 1	103	5.48
Model number 2	210	11.3
